# Local indicators of climate change impacts described by indigenous peoples and local communities: Study protocol

**DOI:** 10.1371/journal.pone.0279847

**Published:** 2023-01-05

**Authors:** Victoria Reyes-García, Santiago Álvarez-Fernández, Petra Benyei, David García-del-Amo, André B. Junqueira, Vanesse Labeyrie, Xiaoyue Li, Vincent Porcher, Anna Porcuna-Ferrer, Anna Schlingmann, Ramin Soleymani

**Affiliations:** 1 Institució Catalana de Recerca i Estudis Avançats, Barcelona, Spain; 2 Institut de Ciència i Tecnologia Ambientals, Universitat Autònoma de Barcelona, Cerdanyola del Vallès, Barcelona, Spain; 3 Centre de Coopération Internationale en Recherche Agronomique pour le Développement, Gestion des Ressources Renouvelables et Environnement, Montpellier, France; 4 Gestion des Ressources Renouvelables et Environnement, Universite de Montpellier, Montpellier, France; CSIR National Environmental Engineering Research Institute (CSIR-NEERI), INDIA

## Abstract

**Introduction:**

In the quest to improve the understanding of climate change impacts on elements of the atmospheric, physical, and life systems, scientists are challenged by the scarcity and uneven distribution of grounded data. Through their long history of interaction with the environment, Indigenous Peoples and local communities have developed complex knowledge systems that allow them to detect impacts of climate change in the local environment. The study protocol presented here is designed 1) to inventory climate change impacts on the atmospheric, physical, and life systems based on local knowledge and 2) to test hypotheses on the global spatial, socioeconomic, and demographic distribution of reported impacts. The protocol has been developed within the framework of a project aiming to bring insights from Indigenous and local knowledge systems to climate research (https://licci.eu).

**Methods:**

Data collection uses a mixed-method approach and relies on the collaboration of a team of 50 trained partners working in sites where people’s livelihood directly depend on nature. The data collection protocol consists of two steps. Step 1 includes the collection of secondary data (e.g., spatial and meteorological data) and site contextual information (e.g., village infrastructure, services). Step 1 also includes the use of 1) semi-structured interviews (n = 20-30/site) to document observations of environmental change and their drivers and 2) focus group discussions to identify consensus in the information gathered. Step 2 consist in the application of a household (n from 75 to 125) and individual survey (n from 125 to 175) using a standardized but locally adapted instrument. The survey includes information on 1) individual and household socio-demographic characteristics, 2) direct dependence on nature, 3) household’s vulnerability, and 4) individual perceptions of climate change impacts. Survey data are entered in a specifically designed database.

**Expected results:**

This protocol allows the systematic documentation and analysis of the patterned distribution of local indicators of climate change impacts across climate types and livelihood activities. Data collected with this protocol helps fill important gaps on local climate change impacts research and can provide tangible outcomes for local people who will be able to better reflect on how climate change impacts them.

## Introduction

There is overwhelming evidence that anthropogenic climate change has a global influence on elements of the atmospheric, physical, and life systems [1, 2], with direct effects on local livelihoods and cultures [3]. However, scientists have a more meagre understanding on how climate change differently impacts local physical and life systems across the globe [4]. Natural scientists acknowledge that downscaling global models to resolutions that are relevant for regional or local level policymaking (e.g., adaptation planning) is challenging due to the uneven data availability across regions and to the uncertainties introduced by downscaling techniques [5]. Moreover, uncertainty in modeling exercises is even larger due to the complex interactions between biophysical systems and human society, which may result in unexpected changes and synergetic impacts [1]. Scientists also argue that a better grasp of climate change impacts requires to couple our understanding of ecological and social dynamics, for which climate change research needs to fully take into consideration impacts on local socioeconomic systems [1, 3, 6, 7].

Different societies are differently affected by climate change, not only because climate change impacts vary along place-specific geographical and ecological characteristics, but also because climate change affects people and societies through specific pathways largely mediated by socio-cultural factors. For example, while sea-level rise is a climate-related phenomenon potentially affecting millions of people living close to sea level, its actual impacts depend not only on biophysical conditions (e.g., magnitude of tidal influences, overall island size and relief) but also on site-specific socioeconomic conditions (e.g., resources to cope with sea-level rise, dependence on fisheries). In sum, the scientific community largely agrees that there is a need to better understand localized climate change impacts and how such impacts are experienced. Yet, in this quest, scientists are challenged by the scarcity of grounded data, which has resulted in climate scientists calling for the exploration of new sources of information [2, 8].

Indigenous and local knowledge systems can contribute to our understanding of local climate change impacts [9–11]. Throughout the world, Indigenous Peoples and local communities (IPLC) with a long history of interaction with their environment have developed intricate and complex knowledge systems (including knowledge, technologies, and forms of organization) that allow them to detect not only changes in local weather and climatic variability [12, 13], but also the direct effects of such changes on the environment on which they depend [14]. Moreover, Indigenous and local knowledge systems are particularly suited to explain how climate change affects local social-ecological systems, livelihoods, and cultures, because of the long-term detailed observation of these systems [2, 15].

Many works have documented Indigenous Peoples and local communities’ first-hand observations of changes in social-ecological systems attributed to climate change [see 9, 10, 14 for reviews]. These works provide rich multi-site qualitative place-based information, but do not consider a common strategy to gather place-specific, yet comparable, knowledge from different locations. Consequently, insights from local knowledge systems continue to lack transferability, integration, and scalability to climate research and policy [10]. To increase transferability, there is a need for a research tool able to draw correspondences between Indigenous and local knowledge’s qualitative and interpretative nature into categories that allow generalization. To allow knowledge integration, there is a need for a tool that allows combining inputs from multi-site place-based research. Finally, to address scalability, there is a need for a community of practice that considers the need to ensure that placed-based information is effectively upscale to climate research [10].

The project Local Indicators of Climate Change Impacts (LICCI, https://licci.eu) was designed to address these challenges through 1) the development of a standardized protocol for the collection of cross-culturally comparable data on local indicators of climate change impacts and 2) the creation of a network of researchers who use this standard protocol. In this paper, we describe the research protocol used for collecting cross-culturally comparable information on direct, observable, localized climate change impacts on elements of the local atmospheric, physical, and life systems and covariates that might affect how this knowledge is distributed within and across societies. We define ‘local indicators of climate change impacts’ (hereafter LICCI) as first-hand observations of environmental change reported by people living in close interaction with a particular environment and attributed–totally or partially- to changes in elements of the atmospheric system (i.e., temperature, precipitation, winds, or seasonality). The overall framework of the project can be found in [10] and the specific tools used for data collection can be found in [16].

## Materials and methods

### Objectives, hypotheses, and research strategy

The LICCI study protocol aims to inventory and identify patterns in reports of local indicators of climate change impacts. Specifically, data collected with this protocol will be used to test hypotheses related to the globally patterned ***i)*** spatial, ***ii)*** socioeconomic, and ***iii)*** demographic distribution of LICCI. Table 1 summarizes the premises, provides examples, and presents the formal hypotheses that guide this work.

**Table 1:** Hypotheses that guide the protocol.

	Premise	Example	Hypothesis
**Spatial patterns**	Climate change impacts are not uniform.	Climate change impacts vary spatially in occurrence and intensity [17].	*H1a*. *Groups living in strongly climate-affected areas will provide higher number and more diverse reports of LICCI than groups living in less climate-affected areas*.
		Climate change impacts vary across biomes and climatic regions.	*H1b*. *Groups living within the same climatic regions will report similar LICCI*.
**Socioeconomic patterns**	Climate change impacts are not equally distributed across the social spectrum.	Climate change impacts affect more intensively people with direct dependence on natural resources [2].	*H2a*. *The higher a household’s direct dependence on natural resources*, *i) the more and ii) the higher severity of LICCI reported*.
		Climate change impacts more intensively people who already face other stressors, such as poverty or lack of access to natural resources [2, 18].	*H2b*. *The higher a household’s vulnerability*, *i) the more LICCI reported and ii) the higher their severity*.
**Demographic patterns**	As other components of local knowledge systems [19], the distribution of knowledge on climate change impacts might be patterned by individual demographic characteristics.	Elders’ reports of changes might have a wider temporal depth than youngsters’ reports [20].	*H3a*. *Elders will i) report more LICCI and ii) a higher severity of their impacts than youngsters*.
		Because their different roles and responsibilities [21], women and men might observe different climate change impacts.	*H3b*. *In each site*, *the similarity on LICCI reports will be higher among women (or among men)*, *than between women and men*.

**Fig 1:**
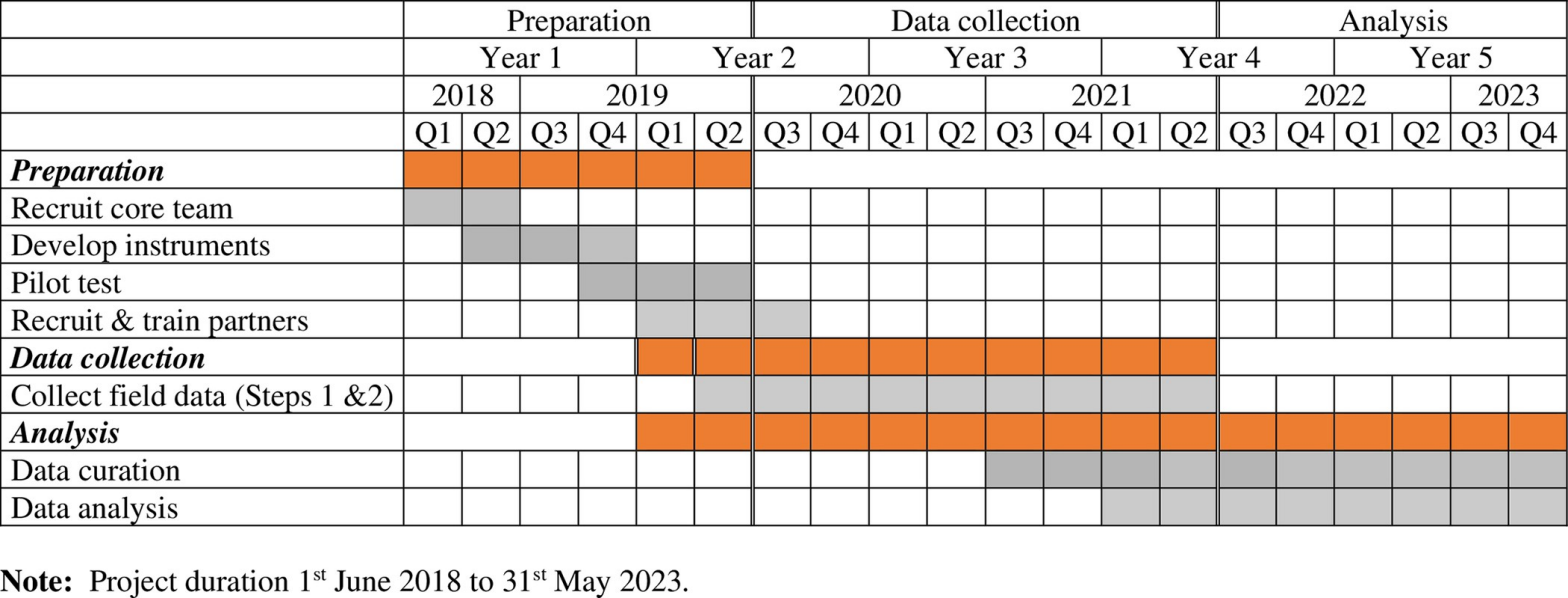
Gantt chart. Development and application of the LICCI protocol. **Note:** Project duration 1St June 2018 to 31’ May 2023.

The LICCI research project started in June 2018 and has a 5-years duration, although the protocol could be used by independent researchers afterwards. Within the project frame, the design, implementation, and use of data collected through this protocol is organized in three phases (Fig 1): The preparation phase (June 2018-December 2019), the data collection phase (January 2020-December 2022), and the data analysis phase (June 2022- June 2023). The estimated time to complete data collection is two person/months, but the overall period of data collection is longer to accommodate different partners’ needs. Due to calendar adjustments derived from the COVID-19 pandemic, data collection and data curation overlapped. The analysis of global data will start once all datasets are curated (December 2022).

### Preparation

The preparation phase includes the development and testing of data collection instruments, the site selection and partners’ recruitment, and partners’ training.

#### Development and testing of data collection instruments

The original development of the data collection instruments was done by the authors. The overall protocol strategy was conceived to rely on a mixed-method approach and the protocol is divided in two steps. Step 1 focuses on the collection of secondary and primary background information, including a list of observations of environmental changes and its drivers, and uses focus group discussions to identify consensus in the information gathered. Step 2 includes a survey to households and individuals to gather data for hypotheses testing.

To improve the overall clarity of the protocol, minimize participant’s fatigue, and avoid sensitive issues that might cause discomfort, we pre-tested all methods in the field. Instruments to be used in Step 1 (i.e., semi-structured interviews and FGDs) were tested with Akha smallholders communities in China, with Betsimisaraka agroforestry farmers in Madagascar, and with Sereer agriculturarists in Senegal. Such testing resulted in 1) a better focus on environmental (vs. global) change, 2) the refinement of our system to code observations of environmental change into indicators of climate change; and 3) a larger consideration of drivers of environmental change other that climate change. Instruments to be used in Step 2 (i.e., individual and household surveys) were tested with Tsimane’ Indigenous Peoples in the Bolivian Amazon and Daasanach agro-pastoralists in Kenya. The second testing also resulted in adjustments of the instrument, including removing sensitive or repetitive questions, or adding questions to capture factors not considering, and reordering or rewording questions to improve the flow.

#### Site selection and partners’ recruitment

**Fig 2:**
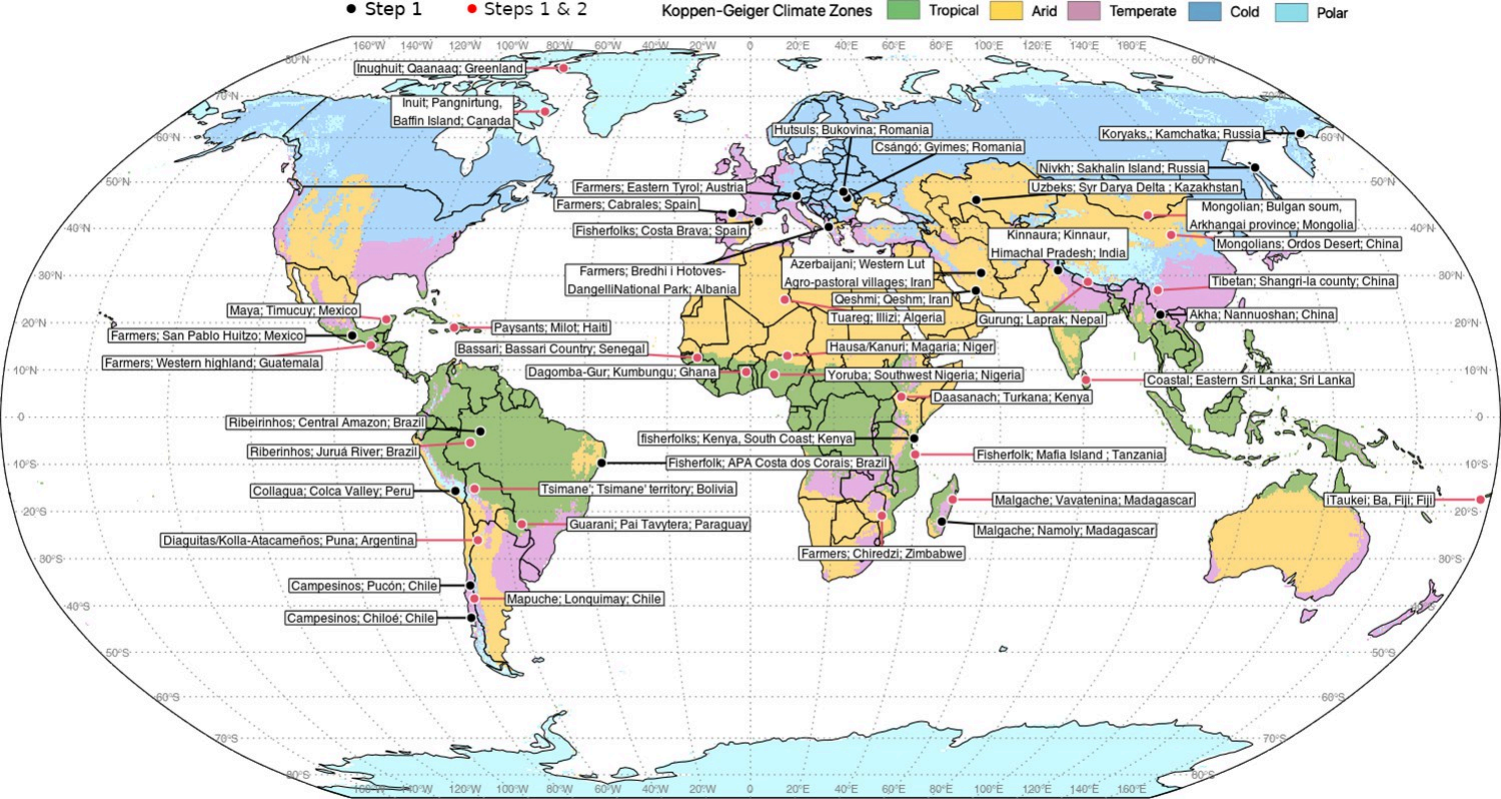
Selected study sites. Sites are displayed across the main different climate types defined by Koeppen-Geiger [22]. Figure elaborated by the authors. Original map from the Natural Earth data set in the public domain (available from https://www.naturalearthdata.com), elaborated with the R libraries maptools [23] and sp [24].

In 2019, the author team worked in the selection of sites where the protocol would be applied. Fig 2 shows the geographical location of the study sites where the protocol has been applied.

The following criteria guided site selection:

1. ***Data availability***: World coverage of weather stations leaves large areas of the world with poor, incomplete, or unreliable weather records, and the literature on local knowledge of climate change impacts is also spatially biased [9, 10]. Hence, we prioritized the selection of sites where instrumental data are deficient and no/few studies on local knowledge of climate change impacts had been conducted.
2. ***Climate type***: To test hypotheses *H1a* and *H1b*, on LICCI spatial distribution, site selection was done to cover the five main different climate types defined by Koeppen-Geiger [22]: tropical, arid, temperate, continental, and polar/cold.
3. ***Predominant livelihood activity***: To test hypotheses *H2a* and *H2b*, about the socioeconomic distribution of LICCI, site selection was done to include sites mainly depending on four livelihood activities (i.e., agriculture, fishing, pastoralism, and foraging).
4. ***Partnership feasibility***: Site selection also included logistical considerations derived from the need to establish an extended network of partners responsible for data collection. To recruit partners, a call was widely circulated. Selection criteria included research experience and previous relations with the proposed study site. The call encouraged South-based researchers to apply.

#### Partners’ training

To minimize interviewer and coder biases during the application of the protocol, all partners participated in a training workshop. Originally, we conducted three one-week face-to-face workshops during 2019 in Barcelona with 48 partners (S1 File). In March 2021, we conducted one additional two-day online workshop to train a set of 10 new partners to substitute partners who had to abandon the project because of the COVID-19 pandemic.

During the workshops, partners were exposed to the rationale of the project and received detailed explanations concerning the application of all the instruments of data collection. Partners also had the opportunity to discuss their schedule for data collection and other practicalities. The workshop included discussions on the code of ethics for the inclusion of Indigenous knowledge in research and the elaboration of a “Local Knowledge Research Agreement” to be discussed and negotiated with communities. The training workshop was also used to discuss issues related to data ownership and sharing (S1 File). Training also included safety considerations related to 1) personal health (e.g., medical contacts, health insurance coverage, vaccination) and safety (e.g., avoiding places locally considered dangerous, avoiding openly exhibiting valuables, driving at night), 2) preparing for emergencies (e.g., allying with a local institution or a trusted local leader, registering with local authorities), and 3) avoiding conflict (e.g., selecting non-conflictive villages). After the training workshop, partners signed a contract with the host institution (Universitat Autònoma de Barcelona) detailing the terms of the agreement (S1 File).

#### Ethical considerations

The research protocol was approved by the Ethics Committee of the Universitat Autonoma de Barcelona (CEEAH 4781) and the LICCI project follows the European Research Council ethical guidelines. Ethical considerations include obtaining Free, Prior and Informed Consent (FPIC) and following Safety and Security guidelines and guidelines for ethical conduct discussed during partners training. All procedures and documents were reviewed by an external and independent Ethics Advisor (Prof. Dr. Michael Schönhuth), who provided continuous reports to the project funders and feedback to the team.

The protocol specifies that before the onset of data collection, all partners need to obtain Free, Prior and Informed Consent (FPIC) from the organizations representing the communities where they aim to work, the villages and the people we work with (S1 File). In the first visit to a village, partners held a meeting to present the research and ask for consent to stay in the village. In the community meetings, partners presented detailed information on the objectives and scopes of the study, the participation of subjects, and the costs and benefits associated with participation, and obtain written consent to participate. In this meeting, partners were also instructed to hold an open discussion leading to a “community engagement protocol” in which the participants had the opportunity to ask in which way they want the information to be returned, or other requirements. Finally, partners also asked for FPIC from each individual.

### Data collection

**Fig 3:**
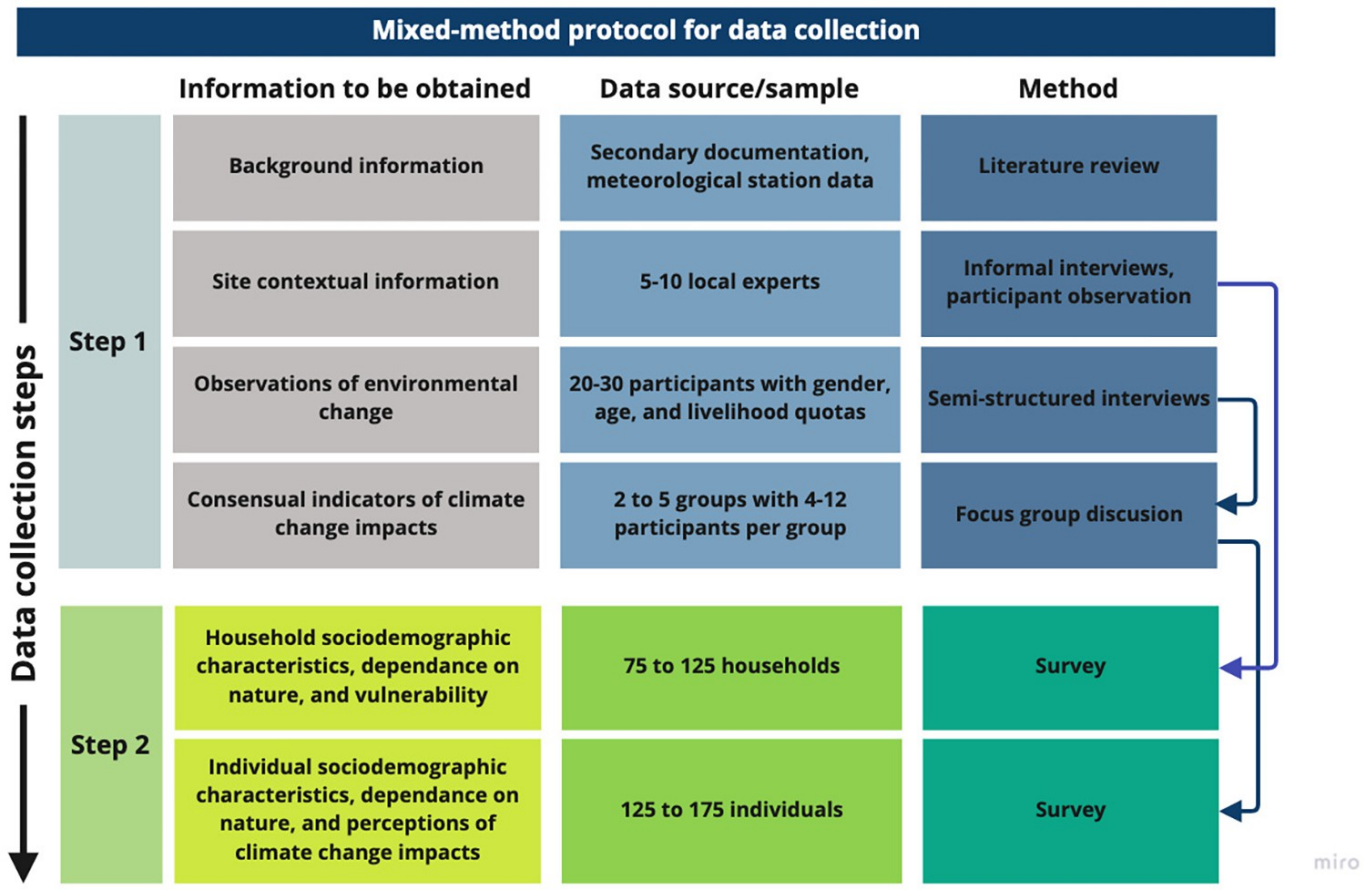
Diagram of data collection protocol displaying the information to be collected, the data source or sample, and the method of data collection. The protocol includes two steps. Step 1 includes the collection of secondary background information, site contextual information, observations of environmental change, and consensual indicators of climate change impacts. Step 2 consists the collection of household and individual characteristics through a survey instrument.

In this section, we describe the sampling strategy to select villages within a specific site and the data collection methods for Steps 1 and 2. The logic of the protocol and the relation between instruments is depicted in Fig 3.

#### Sampling villages

In each site, one partner is responsible for the collection of data across 3–5 villages, defined as the lowest administrative unit in an area and normally under the jurisdiction of a village leader/council. Selected villages should be representative and relatively homogeneous in terms of the environmental and socio-cultural conditions of the site. Villages with particular conditions (e.g., villages with high donor intervention) are to be avoided. To reduce complexity in logistics, targeted villages should have between 20 and 500 households. Villages with more than 500 households should be broken into smaller units and sample one or a few of them.

#### Step 1

In Step 1, partners collect secondary and primary background information from different sources and using different sampling strategies.

Background information to be obtained includes general information (e.g., historical events, protected areas limits) and meteorological data. Background information could be obtained before conducting fieldwork from secondary sources (e.g., literature review, public records, etc.), or while in the field through participant observations and conversation with local officials/knowledgeable individuals. Partners should also obtain geo-referenced data on the location of 1) the study site, villages, neighboring protected areas, and officially recognized Indigenous territories; 2) the closest weather station, airport, and/or harbor/port; and 3) the closest market town and administrative center. Partners will obtain 20-years (minimum) series of data on temperature and precipitation at monthly resolution (minimum) from weather stations within 200 km (maximum) from the study site.

Site contextual information includes the collection of information on local livelihoods and dependence on the natural environment and a timeline of events that are important to the community. Through field observations, secondary sources, and interviews to local experts, partners will collect information about 1) local livelihood activities, including the timing (i.e., seasonal calendar), location, and distribution of these activities, 2) the timing of important events in the recent history of the community (i.e., dated back 80–100 years), and 3) a list of household assets with market value (i.e., fishing nets, machetes etc.), which capture variation in ownership of market assets across households.

Observations of environmental change and their drivers: Partners will use semi-structured interviews to obtain a list of observations of environmental changes. To recruit participants for these interviews, partners will use “quota sampling”. To capture diversity in perceptions of climate change impacts, each partner should select 20–30 participants aiming for a minimum of three interviews per quota across gender (men and women), age (young, a middle aged, and elder), and livelihoods (site dependent). To select people within each quota, partners will rely on key-informants.

Interviews start with the question “Compared to when you were young, what changes in the environment have you noticed?” Additional questions can be then directed to stimulate interviewees to report changes in (a) the atmospheric (e.g., weather/seasons, temperature, rainfall and snowfall, wind, storms); (b) physical (e.g., soil, river, streams), and (c) life system (e.g., wild animals, wild plants, crops, pastures). For each observation of change, partners will also ask about the direction of change (e.g., increase/decrease, earlier/later) and the driver of the change (i.e., “why do you think that this happens? Or “What do you think is the cause of that?”). If the answer to this question is another environmental change, the partner should continue asking about drivers until the respondent could not identify any further driver of the reported observations of change. Note that, to avoid inducing answers, at this stage, the protocol focuses on ‘environmental changes’ in a broad sense and not on ‘climate change’. The attribution to climate change (and/or other drivers) is done *a posteriori*, combining the information on observations of change with that on drivers of change.

Consensual indicators of climate change impacts. Each partner should organize 3–5 *focus group discussions* (FGD) in the study site to discuss information from semi-structured interviews. Between 4 and 12 participants should participate in each discussion. To capture the site diversity in terms of livelihood activities (e.g., farmers, fishers, herders), age, and gender, and to avoid potential conflicts or power imbalances, participants will be selected through convenience sampling.

To prepare for FGD, partners should first classify observations of environmental changes. Following the classification proposed by [14, see also S1 File], partners should start by grouping *verbatim* observations depicting the same environmental change into ‘indicators’ of change. The classification used differentiates between indicators refereeing to changes in elements of ***i)*** the atmospheric (e.g., temperature, rain), ***ii)*** the physical (e.g., water and soil temperature, sea-level rise) and ***iii)*** the life systems (e.g., the morphology, abundance, or distribution of wild and managed/cultivated plant and animal species). For each indicator, partners should also compile information on all the drivers leading to that change. In FGD, partners should discuss the resulting list of indicators of change and their drivers. In particular, partners should ask FGD participants whether they agree on the reported changes and their drivers, focusing on potentially unclear or contradictory information.

Using reports from semi-structured interviews and FGD, partners should also code the level of group agreement in three categories: ‘agreement’ ***i)*** when there is consistency in the report of the indicator and its drivers in semi-structured interviews or ***ii)*** when FGD participants agreed with the indicator and its driver during the discussion; ‘inconclusive’ when observations referring to that indicator are mentioned by less than 10% of people participating in semi-structured interviews and the indicator is not discussed in FGD; and ‘disagreement’ when observations referring to the same indicator report different directions or drivers and/or there is no agreement on the indicator and its drivers during FGD.

The consensual list of LICCI for a site should include all indicators for which there is agreement and that are reportedly driven (directly or through cascading effects) by changes in elements of the atmospheric system. The participatory, collective, and iterative nature of the process ensures that the final list of LICCI in a site reflects the group *social memory*.

#### Step 2

Step 2 consists of the application of a structurally identical survey, but with site-specific questions derived from information collected in Step 1. The survey includes questions directed to the household and questions directed to the individuals (see [16] for the exact survey questions). To select households (between 75 and 125), partners should use *simple random sampling*, drawing households from a local census. Household questions can be answered by anyone who is considered a household head, defined as a person who–by formal or informal rules- can take decisions on household’s labor and income. Within each household, partners should use *convenience quota sampling* among household heads to independently answer individual questions. Individuals (between 125 and 175) should be approximately evenly distributed across gender and age categories.

The survey will include four sections.

1. *Individual and household socio-demographic characteristics* including household composition and information from household members (i.e., level of formal education, occupation, migratory status), history of family belonging to the region, access to meteorological information, and individual’s subjective wellbeing and climate change awareness.
2. *Individual and household dependence on natural resources* will be proxied using the ´pebble distribution method´ [25, 26], in which respondents will be asked to distribute a given number of points (´pebbles´) across different livelihood activities practiced locally, placing pebbles based on the time invested (individual level) and income obtained (household level) from each activity. Livelihood activities include natural resource-related activities as well as activities unrelated to natural resources.
3. *Household’s vulnerability*: We draw on the sustainable rural livelihoods approach [27] and proxy vulnerability as a factor of (lack of) access to the five capitals (i.e., financial, physical, human, social, and natural capital) that allow households to pursuit their livelihood strategies. To assess *financial capital*, we ask questions about income from different sources, savings, and credit. To assess *physical capital*, we ask about ownership of 10 market assets, housing material, and access to communal assets (e.g., access to roads or local infrastructure). To assess *human capital*, we ask about household composition, formal education, number of languages spoken, and local knowledge. To assess *social capital*, we ask about membership to associations, social relations inside and outside the village, and perceptions of trust and cooperativeness. Finally, to assess access to *natural capital*, we ask about access to water and local food, land and livestock ownership, and access to communal resources (e.g., common forest, pastures, fisheries) for both sustenance and income generation.
4. *Individual perceptions of climate change impacts*: Partners will use the site-specific list of consensual LICCI to randomly select 15 LICCI to be included in the survey. For each LICCI, we will ask survey respondents to report a) whether they have observed the change (i.e., *indicator perceived)*, 2) whether that change has any noticeable (positive or negative) impact on their livelihood (i.e., *impactful indicator perceived)*, and 3) the *severity of the impact*. Adapting a method developed in medical research [28], to measure the severity of a LICCI, we will ask whether the impact affected the person 1) a lot, 2) a little, 3) not at all.

As some survey questions are site-specific a draft version of the survey should be pre-tested with 10 informants. The draft version of the survey should include 30 LICCI from the consensuated list and 15 commercial items displaying variation. Results from the test will be used to select the assets and LICCI to be used in the final survey to be applied in a site.

### Data analysis

*To test hypotheses on spatial patterns*, we will rely on site-specific lists of LICCI and geographical information. We will elaborate a map displaying all LICCI in the inventory and use open-source platforms to visualize the geo-referenced database of reported impacts and use spatial matching techniques to search for patterns across different *i)* climate-affected areas [29] (*H1a*) and *ii)* Köppen climatic areas [17] (*H1b*).

*To test hypotheses on socioeconomic patterns*, we will use survey data. We will aggregate responses to survey questions to create three dependent (i.e., *number of indicators perceived*, *number of impactful indicators perceived*, *severity of impacts)* and two explanatory variables (i.e., *direct dependence on natural resources* and *vulnerability*) to be used in multivariate regressions. The *number of indicators* and *impactful indicators perceived* correspond to the sum of LICCI from those included in our survey instrument reported by the interviewed person. The score of *perceived severity* will be a composite measure created from data on the perceived severity of the various LICCI included in the survey. The number of pebbles allocated to different livelihood activities will be used as a measure of the importance of that activity to the respondent and the number of pebbles allocated to natural resource-related activities will be used as a measure of household *dependence on natural resources*. We will create an index of household *vulnerability* by normalizing the measures of the different indicators of household’s relative access to the five asset types defined and aggregating them into a single measure. To estimate how the *number of indicators*, *impactful indicators* and *severity of impacts perceived* vary across households depending on their direct dependence on natural resources (*H2a*) and vulnerability (*H2b*), we will use regression analysis. Specifically, we will model each of the three dependent variables as a function of *direct dependence on natural resources* and *vulnerability* while controlling for additional variables that might affect the studied relation (e.g., site). As data to test H2 are naturally hierarchical in structure, with households nested within villages, and villages nested within climatic areas, we will consider the multilevel structure of model variance by fitting mixed-effect models. For the statistical analysis, we will use the package *nlme* [30] in R statistical software version 4.2.1.

*To test hypotheses on demographic patterns*, we will use the exact same dependent variables and a similar analytical approach. In this analysis, we will use as main explanatory variables the *age* or *position in the life cycle* (i.e., young, adult, or elder), as aggregated measures of age might improve the reliability of estimates *(H3a)*, and the *sex* of the informant (*H3b*).

### Data management

Partners submit data through a web application specifically developed for this study protocol and accessible for registered partners (S1 File). The application stores data in the browser of the device used, which can then be submitted to a particular website following a set of guidelines (S1 File). To strengthen intercoder reliability and minimize data coding and submission errors, during the data collection period (2020–2022), authors closely worked with partners to clarify doubts in data collection, coding, and submission.

The LICCI project participates in the Horizon 2020 Open Research Data pilot and follows the ‘FAIR data principles’, for which it will make the project’s research data findable, accessible, interoperable, and re-usable. Procedures for the management of data, from collection to use after the project ends are outlined in the project’s Data Management Plan (DMP), which includes data and metadata standards for use of data, policies for access and sharing, policies for re-use and distribution, and plans for data archiving and preservation.

Data collected with this protocol will only be made open if study participants agree on it. Results from Step 1 of the project will be made available only in aggregated form. Anonymized survey data will be made available with the publication of research results, also in an aggregated format. All data will be stored on and available from Harvard’s Dataverse. To describe the underlying data, metadata with sufficient detail will be created to be intelligible for other users. The LICCI metadata will follow a generalized scheme in the Dataverse including, among others, title, creator(s) and contact person(s); date; version; location; contributor, e.g., funding body, including the grant agreement number; data format(s); keywords; identifiers (DOI and url); access rights (license(s)), and other labels, including labels on the particularities for access and use of cultural heritage data that is digitally circulating outside community contexts to respect local data sovereignty [31]. The LICCI team applies an embargo period of 24 months from data submission to data release to ensure that the partner collecting the data has a first right to publication. In June 2024, the data will be made public on Dataverse after a thorough anonymization process to ensure no sensitive information is released, considering data sensitivity from an individual and a cultural perspective.

## Discussion

This section discusses 1) the main opportunities of using this protocol and the potential impact of data collected using it and 2) the main challenges of using this protocol and the limitations of data collected using it.

### Opportunities and potential impact

Researchers are challenged to provide robust and implementable information on climate change impacts, particularly at the local scale. The protocol presented here provides an opportunity to address this challenge for several reasons. First, the inventory of LICCI will improve our understanding of the localized responses of the physical and life systems to climate change. Importantly, such data refers to remote areas, difficult to extensively sample with traditional field methods. The data collected through this protocol can complement research based in other sources of data (e.g., weather stations, remote sensing, biological surveys) to help define more precisely the linkages between climate change and the way it is reflected in local systems. The potential to fill data gaps regarding how climate change impacts the biophysical world directly answers the call of the Intergovernmental Panel on Climate Change (IPCC) to develop the evidence base for the potential contribution of local knowledge to climate research [32].

Second, data collected through this protocol will improve our understanding of the impacts of climate change on local livelihoods, from changes in cropping patterns and damages related to extreme events [33], to disease outbreaks or to the increase in conflicts over dwindling resources [34]. While responding to climate change demands adjusting to weather and climate changes; identifying risks, making decisions on how to respond, and implementing such decisions are all mediated by socioeconomic and cultural factors [3]. The information collected through this protocol will expand our knowledge on climate change impacts on local livelihoods and thus improve our understanding of the human dimensions of climate change and guide policies attempting to mitigate its immediate impacts.

Third, the involvement of local communities in data collection holds the potential to increase local agency for adaptation [35]. Local people will actively participate in identifying climate change impacts and the resulting information will be reported back to them. This process increases the likelihood that local people take ownership over the process and results, potentially empowering them in crafting, determining, and adopting viable strategies for coping with and adapting to climate change immediate impacts on their livelihood.

### Challenges and limitations

The use of the protocol faces three main challenges and one important limitation. First, the use of the protocol faces the challenge to mobilize Indigenous and local knowledge in a structured and organized way, while -at the same time- defying the prevailing technical approach to document information from other knowledge systems which seldom captures their intricacies [15]. To overcome the challenge, we selected partners with previous or planned long-term contact with the study site and train them to collect information that reflects group social memory. We recommend that further applications of this protocol continue to rely on researchers with long-term involvement with local communities or on researchers-local communities’ partnerships to limit misinterpreting Indigenous and local knowledge.

The second challenge is to adequately capture the temporal dimension of change, as neglecting it might lead to conflate climate change and weather variability. For example, in a case study derived from this work [33], researchers found that Tuareg participants reported the impact of rain and temperature irregularities and severe drought events on their pastoral and semi-pastoral livelihoods. Paradoxically, they did not explicitly report decadal trends in the frequency of extreme events. The differential perception of climate change impacts across time scales can have important implications for undertaking climate change adaptation measures. To address the challenge, we designed the protocol to capture information encapsulated in the group’s *social memory*, or the cumulative and shared knowledge encompassing long-term, intergenerational observations of immersive experience in a particular place [36]. Since social memory provides a more accurate temporal assessment than individual observations, we recommend that future work continues developing research based on social (rather than individual) information.

A third challenge of the use of this protocol refers to the need to be adapted to different context and social-ecological systems. To overcome the challenge, we put large emphasis in designing a flexible protocol. This strategy also increases the diversity of outcomes from the project, as reflected in the first case studies published by partners who have used data collected through Step 1 [33, 34, 37]. We also invested in the training of partners to understand the concepts behind the questions, so they could use their experience to locally adapt the protocol. For example, due to the COVID-19 pandemic, in many sites was not possible to conduct FGD to maintain social distancing. Several partners modified the protocol to validate the consensual nature of indicators collected in semi-structured interviews through additional interviews to other informants. Given the large carbon impact of our training strategy [38], we have created a number of written and online training materials, as well as a Massive Open Online Course (MOOC) about climate change and Indigenous peoples and local communities (S1 File). We recommend the use of these online materials to guide and train researchers aiming to use this protocol in the future. The use of the online training materials will continue to ensure comparability across datasets, while minimizing carbon impact.

The main limitation of this research relates to the ability to discern whether reported changes in elements of the atmospheric, physical, and life systems can be really attributed to climate change, as complexity in such systems and confounding drivers of change (e.g., geological processes, land-use change, or human demography shifts) make attribution difficult [8]. Indeed, researchers increasingly recognize the synergistic effects of many drivers of change [1]. While such problem is common to any research on climate change impacts, it seems to be aggravated when assessing localized impacts, the focus of this work. We cannot fully tackle this limitation, but we notice that for many Indigenous Peoples and local communities the effect of different drivers of change are actually intertwined.

## Supporting information

S1 File Online resources associated to the LICCI project. (DOCX)

S2 File Online resources associated to the LICCI project. (DOCX)
