# Supplementary material for: Local indicators of climate change impacts described by indigenous peoples and local communities: Study protocol
Source: PLoS One. 2023 Jan 5;18(1):e0279847. doi: 10.1371/journal.pone.0279847 (PMC9815565; doi:10.1371/journal.pone.0279847)
Supplement: S2 File — (DOCX) [file pone.0279847.s002.docx]

**S1 File. Online resources associated to the LICCI project**

The LICCI project adheres to the principles of Open Science, follows the ‘FAIR data principles’ and participates in the Horizon 2020 Open Research Data pilot. Throughout the project, the team has done a large effort to document the scientific process and, consequently, there are many materials already available. Below we provide a list of already available online resources.

- LICCI project webpage: <https://licci.eu/>
- LICCI project twitter: <https://twitter.com/LICCI_ERC>
- Master Manual for data collection: <https://doi.org/10.6084/m9.figshare.11513511.v3>
  - Publication policies, pp. 61
  - Partners’ contract, pp. 66
  - Ethics procedures, pp 20-26
- LICCI App for data collection: <https://licci.eu/app/>
- Guidelines for the use of LICCI App: <https://licci.eu/app-guidelines/>
- Data Management Plan: <https://licci.eu/current_dmp>
- Training workshops: <https://www.youtube.com/playlist?list=PLUiey9Ly1EHvuctsXij0wBF_ifWLrt3Xb>
- MOOC: <https://www.coursera.org/learn/climate-change-indigenous-communities>
- Project data will be available at <https://dataverse.harvard.edu/>
